# Caffeic Acid as a Promising Natural Feed Additive: Advancing Sustainable Aquaculture

**DOI:** 10.3390/biology14091160

**Published:** 2025-09-01

**Authors:** Nguyen Dinh-Hung, Luu Tang Phuc Khang, Suwanna Wisetkaeo, Ngoc Tuan Tran, Lee Po-Tsang, Christopher L. Brown, Papungkorn Sangsawad, Sefti Heza Dwinanti, Patima Permpoonpattana, Nguyen Vu Linh

**Affiliations:** 1 Aquaculture Pathology Laboratory, School of Animal & Comparative Biomedical Sciences, The University of Arizona, Tucson, AZ 85721, USA; dinhhung@arizona.edu; 2 Aquatic Biotechnology Laboratory, Department of Animal and Aquatic Sciences, Faculty of Agriculture, Chiang Mai University, Chiang Mai 50200, Thailand; khang_luu@cmu.ac.th (L.T.P.K.); suwanna_w@cmu.ac.th (S.W.); 3 Institute of Marine Sciences, Shantou University, Shantou 515063, China; tranntts@gmail.com; 4 Department of Aquaculture, National Taiwan Ocean University, Keelung 202, Taiwan; leepotsang@email.ntou.edu.tw; 5 FAO World Fisheries University Pilot Programme, Pukyong National University, Busan 47340, Republic of Korea; brownchristopher38@gmail.com; 6 School of Animal Technology and Innovation, Institute of Agricultural Technology, Suranaree University of Technology, Nakhon Ratchasima 30000, Thailand; papungkorn@sut.ac.th; 7 Department of Aquaculture, Faculty of Agriculture, Sriwijaya University, Inderalaya 30662, Indonesia; sefti.heza@unsri.ac.id; 8 Department of Agricultural Science and Technology, Faculty of Innovative Agriculture, Fisheries and Food, Prince of Songkla University, Surat Thani Campus, Surat Thani 84000, Thailand

**Keywords:** aquafeed additives, disease resistance, immunomodulatory, sustainable aquaculture, phyto-additives

## Abstract

Caffeic acid (CA), a plant-derived phenolic compound, is emerging as a promising natural feed additive for sustainable aquaculture. Its growth-promoting, immunomodulatory, and antimicrobial activities suggest utility as an alternative that diminishes antibiotic use in fish farming. Evidence across multiple species indicates improvements in innate immune responses, enhanced antioxidant capacity, and increased survival during pathogen challenge. Nevertheless, adoption remains limited by unresolved questions regarding optimal inclusion levels, species-specific physiological responses, interactions with other dietary components, and effects on the gut microbiota. This review synthesizes current research on CA, critically evaluates its functional roles in aquaculture, and assesses its relevance to sustainable production. Priorities for future work include elucidating mechanisms of action, conducting cross-species dose–response studies, standardizing dosing protocols, clarifying microbiome effects, and evaluating economic feasibility for large-scale use. Addressing these gaps will be essential to realize the full potential of CA as a functional feed additive in sustainable aquaculture systems.

## Simple Summary

As global aquaculture expands, the need for safe, sustainable alternatives to antibiotics has become increasingly urgent to safeguard the health and productivity of aquatic animals. Caffeic acid (CA), a naturally occurring plant polyphenol, has emerged as a promising candidate owing to its multifunctional bioactivity. This review provides a comprehensive evaluation of CA’s potential applications in aquaculture, synthesizing evidence that CA enhances immune responses, reduces inflammation, exerts antimicrobial effects, and improves overall fish health. Although the research base is still developing, CA appears to act through distinct biological mechanisms that may differentiate it from other plant-derived feed additives. This review also highlights key knowledge gaps, including the need to define optimal dosing strategies, understand species-specific responses, investigate impacts on the gut microbiota, and assess cost-effectiveness for commercial-scale use. Addressing these priorities will lay the groundwork for future studies and support the development of sustainable functional-feed strategies that diminish antibiotic reliance in aquaculture.

## 1. Introduction

**Figure 1:**
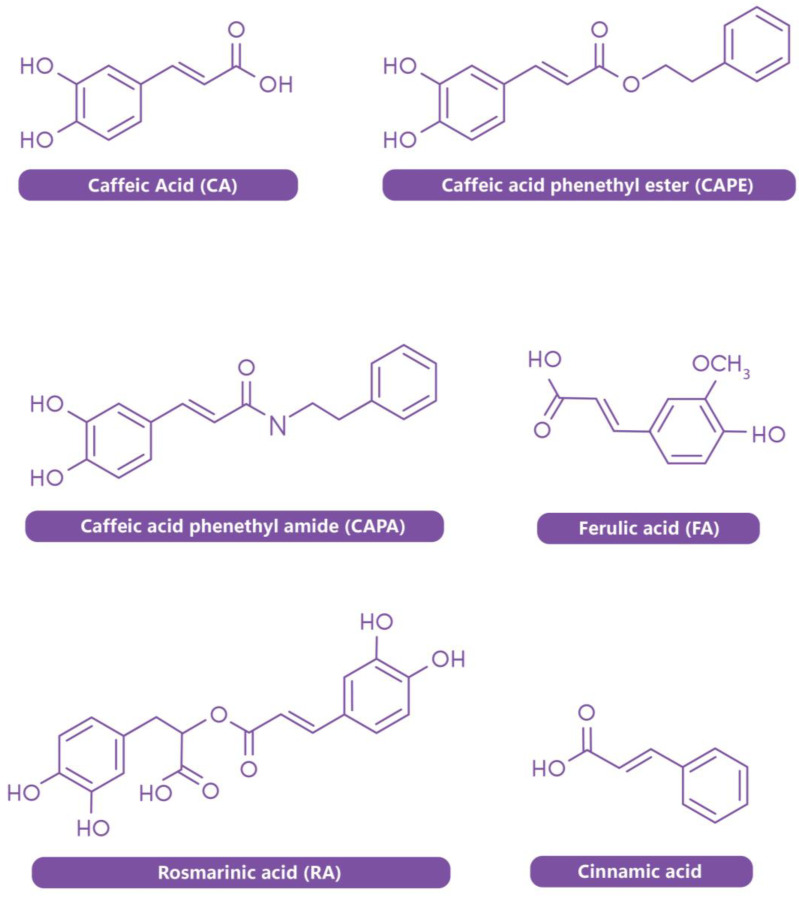
Caffeic acid and its derivatives.

Aquaculture is recognized as the fastest-growing sector of global food production, serving as a critical solution to meet the rising demand for aquatic foods driven by an expanding global population [1]. As wild fisheries continue to decline due to resource overexploitation [2,3], aquaculture has become an essential solution for bridging the widening gap between supply and demand, offering a more sustainable and reliable source of high-quality nutrition [4,5]. However, the intensification of aquaculture systems has introduced several sustainability challenges, including dependence on fishmeal-based diets, environmental degradation, and the proliferation of antibiotic-resistant bacteria resulting from the overuse of antimicrobials [6,7,8]. To address these challenges, there is growing interest in the development and application of functional feed additives—such as probiotics, prebiotics, immunostimulants, and plant-derived bioactive compounds—that enhance animal health while reducing environmental and chemical burdens [9,10,11]. Among these, caffeic acid (CA), a naturally occurring phenolic acid widely distributed in fruits, vegetables, and medicinal herbs, has emerged as a promising candidate [12,13]. Caffeic acid possesses multiple biological activities, including potent antioxidant, anti-inflammatory, and antimicrobial effects, and has demonstrated the ability to modulate reactive oxygen species (ROS) and enhance immune function in aquatic animals [14,15]. Compared to other phyto-additives such as flavonoids and tannins, CA is distinguished by its structural simplicity, cost-effective extraction, and versatility in dietary formulation applications [16], making it particularly attractive for large-scale aquaculture. These features position CA as a practical and sustainable alternative to more complex or less bioavailable compounds. Beyond CA itself, its structural derivatives (Figure 1), including chlorogenic acid, rosmarinic acid, caffeic acid phenethyl ester (CAPE), ferulic acid, cinnamic acid, and caffeic acid phenethyl amide (CAPA), exhibit modified or enhanced biological functions. For example, chlorogenic acid (an ester of CA and quinic acid) exhibits robust antioxidant and antimicrobial properties, contributing to improved performance and reduced oxidative stress in fish [17]. CAPA and CAPE, derived via amide and ester bonds, respectively, have demonstrated strong immunomodulatory and anti-stress activities [18]. Similarly, rosmarinic acid, another CA ester, shows potent anti-inflammatory effects. These derivatives further broaden the scope of CA-based compounds as functional ingredients in aquaculture nutrition. Empirical studies have shown that dietary supplementation with CA can promote growth, stimulate immune responses, and increase resistance to pathogens across a range of cultured aquatic species (Table 1). Furthermore, its application aligns with the broader goals of sustainable aquaculture by potentially mitigating environmental impacts and reducing antibiotic dependency.

This review critically examines the mechanisms of action, nutritional advantages, and practical applications of CA and its derivatives in aquaculture, highlighting their roles as sustainable alternatives to antibiotics and synthetic chemical additives. Despite encouraging evidence, several critical gaps remain. These include the need to understand species-specific physiological and immunological responses, to elucidate the molecular and cellular pathways underlying CA’s bioactivity, to determine its modulatory effects on gut microbiota, and to assess its cost-effectiveness in commercial aquaculture systems. Addressing these knowledge gaps will require multidisciplinary, species-targeted research approaches, including omics-based investigations, controlled feeding trials, and techno-economic evaluations. Advancing our understanding of CA’s functional roles will support its rational incorporation into feed strategies and contribute to the long-term sustainability and resilience of global aquaculture.

## 2. Sources of CA and Its Derivatives

CA and its derivatives are abundant in various natural sources, including fruits, coffee-related products, teas, and other plant materials (Table 2). Chlorogenic acid, a major precursor of CA, is present in fruits such as apricots, cherries, plums, and peaches at concentrations ranging from 50 to 500 mg/kg [69]. Significant amounts of CA are also found in coffee beans, potatoes, apples, and tobacco leaves. Coffee-related products serve as notable reservoirs, with green coffee beans containing 49.57 mg/g dry weight (dw) [70], defective coffee beans at 0.038 g/g dw [71], and coffee pulp at 2 mg/g dw [71]. Additionally, green and black teas provide CA concentrations ranging from 0.30 to 0.36 mg/g [72]. The extraction of CA typically involves solvent-based methods using ethanol, methanol, or water, followed by purification techniques such as liquid–liquid extraction and high-performance liquid chromatography [73].

The efficiency of extraction is influenced by solvent selection, temperature, duration, and pH levels [79]. Environmental factors during plant growth, such as soil quality, climate conditions, and harvest timing, also affect CA content [69]. Post-harvest storage conditions, including temperature and humidity, further impact the stability and functionality of CA and its derivatives [80]. High-purity CA exhibits potent biological activities, including antioxidant, antimicrobial, and anti-inflammatory properties, which are essential for its application as a functional feed additive in aquaculture. Optimizing extraction methods and understanding factors affecting CA’s purity and functionality are crucial for enhancing its efficacy in aquafeeds and ensuring its sustainable use in aquaculture systems. Thus, CA’s scalability and cost-effectiveness, supported by its availability from agricultural byproducts and efficient synthesis methods, enhance its viability for widespread application.

## 3. Applications of CA and Its Derivatives in Aquaculture

### 3.1. Applications of Caffeic Acid

Geographically, research on the application of CA in aquaculture spans Asia, the Middle East, and Europe, reflecting its broad potential as a feed additive, health promoter, and preservative. In Asia, particular attention has been directed toward CA derivatives. For example, a recent study in China demonstrated that dietary supplementation with CAPE in grass carp (*Ctenopharyngodon idellus*) mitigated the adverse effects of a high-carbohydrate diet. CAPE enhanced adipocyte hyperplasia, increased lipolysis and β-oxidation in adipose and hepatic tissues, improved glucose uptake and utilization, and reduced inflammation and hepatic steatosis [28]. These findings support the potential use of CA compounds to facilitate more cost-effective, carbohydrate-rich diets in large-scale aquaculture operations. In Europe, research has primarily focused on CA’s role in improving feed quality and post-harvest preservation. Spanish investigators reported that ex vivo application of CA to fish muscle significantly delayed lipid oxidation by regenerating endogenous antioxidants such as vitamin E [81]. Meanwhile, studies in Turkey have emphasized its antimicrobial potential; for instance, Tapan et al. [82] demonstrated that CA inhibited several pathogenic bacteria of fish in vitro, underscoring its promise as a natural alternative to antibiotics in aquaculture. Collectively, these research highlight the diverse applications of caffeic acid in aquaculture, as a growth promoter, immunostimulant, antimicrobial agent, and post-harvest preservative.

CA has been studied extensively as a multifunctional feed additive and post-harvest treatment due to its potent antioxidant, immunostimulatory, and preservative properties. For example, dietary CA supplementation at 5–10 g/kg significantly enhanced the activities of antioxidant enzymes such as SOD, CAT, and GST, while improving growth performance and immune responses in beluga sturgeon (*Huso huso*) [20,83]. Similarly, Nile tilapia fed 5 g/kg CA exhibited elevated activities of SOD, CAT, and GPx, alongside improvements in blood biochemistry, gut morphology, and resistance to pathogenic challenge [22]. In addition to dietary applications, CA has also been utilized as a dipping agent or incorporated into chitosan-based coatings to extend the shelf life and preserve the quality of fillets from species such as sea bass and pompano by reducing lipid oxidation, microbial contamination, and protein degradation [31,32].

### 3.2. Applications of CA Derivatives

CA derivatives, most notably CAPE, chlorogenic acid (CGA), metal–CA complexes, and CA-grafted polymers, have attracted increasing interest in aquaculture due to their diverse bioactivities and functional potential. CAPE has been extensively evaluated in grass carp (*C. idellus*) under metabolic stress conditions. Supplementation of high-fat diets with CAPE at 0, 200, 500, or 800 mg/kg feed significantly improved feed conversion ratios, promoted adipocyte hyperplasia and healthy adipose tissue remodeling, alleviated hepatopancreatic steatosis and downregulated pro-inflammatory cytokines (e.g., IL-1β, TNF-α) in the adipose tissue, hepatopancreas, and muscle. Additionally, CAPE reduced serum levels of lactate dehydrogenase (LDH), alanine aminotransferase (ALT), aspartate aminotransferase (AST), and low-density lipoprotein cholesterol (LDLC) [27]. When incorporated into a high-carbohydrate diet at the same dose range, CAPE activated peroxisome proliferator-activated receptor gamma (PPARγ) in adipose tissue, enhanced lipolysis and β-oxidation, promoted glucose uptake-related gene expression, and mitigated hepatic steatosis and inflammation, leading to reduced levels of ALT, AST, glucose (GLU), lactate dehydrogenase (LD), LDLC, and triglycerides (TG) [28]. Beyond metabolic regulation, CAPE has demonstrated cytoprotective effects in zebrafish (*Danio rerio*) larvae exposed to ototoxic neomycin (125 µM). Co-treatment with CAPE (50–1000 µM) dose-dependently preserved neuromast hair cells, prevented mitochondrial damage, and reduced apoptosis. These protective effects were most pronounced at 500 µM within a one-hour exposure window [29], suggesting novel applications of CAPE in sensory cell preservation and prompting further investigation into its mechanisms of action.

Chlorogenic acid (CGA), an ester of caffeic and quinic acids, has been evaluated in both fish and crustacean models. In Amur ide (*Leuciscus waleckii*), dietary CGA (0.04%) alleviated LPS-induced intestinal injury by upregulating tight junction proteins (ZO-1, occludin, and claudin), enhancing antioxidant enzyme activities (GSH-PX, CAT, and SOD), and suppressing the NF-κB and caspase signaling pathways [33]. In Asian swamp eel (*Monopterus albus*), CGA supplementation (250–750 mg/kg) improved growth performance, increased serum HDL and antioxidant enzyme levels, and reduced TG, GLU, LDL, and malondialdehyde (MDA) concentrations [34]. Similarly, in blackspotted croaker (*Protonibea diacanthus*), CGA (100–1600 mg/kg) reduced muscle oxidative stress and inflammation while enhancing texture and collagen deposition [35].

Rosmarinic acid (RA), a di-caffeoyl ester, has also demonstrated immunostimulatory and growth-promoting effects. In common carp (*Cyprinus carpio*), 600 mg RA/kg feed, with or without *Bacillus subtilis*, improved growth, digestive enzyme activity, immune parameters (e.g., lysozyme, ACH50), and survival following *Aeromonas hydrophila* challenge [66]. In *Carassius auratus*, 400–800 mg/kg RA dose-dependently enhanced growth and upregulated immune-related gene expression [67]. In rainbow trout (*Oncorhynchus mykiss*), co-supplementation with 1–3 g RA/kg and *Lactobacillus rhamnosus* yielded synergistic improvements in growth, antioxidant capacity, and stress tolerance [68].

Ferulic acid (FA), a methoxylated analog of CA, has been shown to possess antioxidant, anti-inflammatory, and neuroprotective properties. In blunt snout bream (*Megalobrama amblycephala*), dietary FA (100–200 mg/kg) enhanced growth, feed efficiency, and the activities of CAT, SOD, and GPx, while downregulating inflammatory and endoplasmic reticulum (ER) stress-related genes (e.g., IL-6, NF-κB, and caspases) [51]. In common carp (*C. carpio*), 200 mg/kg FA conferred neuroprotection against pesticide exposure by activating the Nrf2/Keap1 pathway and suppressing apoptosis markers such as Bax and caspase-3 [52,53,54]. However, direct comparative studies between FA and other CA derivatives like CAPE or CGA remain limited.

Cinnamic acid (CinA), the simplest CA analog, has been studied primarily for its effects on gut health and immunity. In Nile tilapia (*Oreochromis niloticus*), CinA supplementation improved FW, FI, SGR, and protein retention, alongside elevated antioxidant and digestive enzyme activities and upregulated immune gene expression [23]. In rainbow trout, CinA (0.25–1.5 g/kg) lowered intestinal pH, serum triglycerides, and hepatic enzyme levels (AST and ALT), while upregulating hepatic antioxidant genes (SOD and GST) [49]. When co-administered with *B. subtilis* (25–150 mg/kg), CinA enhanced respiratory burst, phagocytosis, myeloperoxidase (MPO) activity, and resistance to *Yersinia ruckeri* infection [50].

Emerging non-dietary applications of CA derivatives further broaden their relevance to aquaculture. For environmental remediation, CA complexed with Cr(III) or Pb(II) enabled efficient removal of heavy metals via flotation, producing effluents with LC_50_/EC_50_ values exceeding 100 mg/L in carp (e.g., *C. carpio*, *Daphnia magna*, and *Selenastrum capricornutum*), thereby demonstrating potential for wastewater management in aquaculture [30]. In post-harvest preservation, carbodiimide-mediated grafting of CA onto chitosan (CS-g-CA) significantly improved the quality of pompano (*Trachinotus ovatus*) fillets. CS-g-CA coatings (1%) reduced texture softening, inhibited protein degradation, preserved myofibrillar microstructure, and enhanced sensory properties, especially when combined with ultrasonic treatment [31,32]. These findings highlight a growing trend toward the development of CA-based biocompatible materials aimed at improving aquaculture biosafety and product quality.

### 3.3. Challenges and Gaps in Applying CA and Its Derivatives in Aquaculture

Stability during feed processing and storage remains a key practical consideration for the application of natural additives in aquaculture. CA is relatively stable; for example, one feeding trial demonstrated that CA and gallic acid concentrations remained unchanged after 70 days of storage at elevated temperatures (28–38 °C) [84]. In contrast, FA is less thermally stable and undergoes significant degradation during high-temperature processing [85]. Similarly, CGA, a CA derivative, is prone to isomerization upon heating [86]. Essential oils (EOs) are particularly vulnerable due to their volatility and susceptibility to oxidation unless stabilized, commonly through microencapsulation techniques [87,88]. Among all additives, probiotics are the least stable, as they consist of live microorganisms whose viability is highly sensitive to temperature, humidity, and pH. Their functional integrity typically depends on protective formulations and stringent storage conditions [89,90]. Thus, from a stability perspective, CA and other phenolic acids generally exhibit greater resilience than heat-sensitive compounds like FA, EOs, or probiotics, provided that feed storage practices are optimized. Nevertheless, all natural additives require appropriate formulation strategies, such as drying or encapsulation—and temperature control to ensure shelf life and efficacy.

Economic feasibility is another critical factor shaping the selection and adoption of feed additives. Many phenolic compounds, including CA, can be extracted from low-cost plant-based or agro-industrial by-products such as olive mill waste or spent coffee grounds, which helps reduce raw material expenses. However, the overall cost-effectiveness also depends on extraction, purification, and incorporation into aquafeeds. A recent review highlighted the need for more efficient production technologies to ensure the commercial viability of natural additives [91]. In comparison, essential oils often require large volumes of plant biomass and distillation processes [92,93], resulting in higher per-unit costs of active compounds. Similarly, probiotics involve significant expenditures related to microbial culture, fermentation, drying, and cold-chain storage. By contrast, powdered phenolics such as CA or FA can be manufactured at scale from agricultural waste streams, potentially making them more economically viable. Given that effective inclusion rates for CA are relatively low, typically a few grams per kilogram of feed, the added cost per ton of formulated feed is modest. In many cases, this incremental cost may be offset by measurable improvements in growth performance, immune function, and disease resistance. For example, Yılmaz [22] reported that 5 g/kg CA supplementation provided disease protection comparable to antibiotic treatment, suggesting a favorable cost–benefit profile. Furthermore, Hu et al. [91] projected growing interest in organic acids like CA due to their sustainable health benefits and low risk of adverse effects. Although comprehensive economic assessments are still limited, current evidence suggests that CA is a promising and cost-effective feed additive in diverse aquaculture systems.

Despite these promising attributes, several research gaps hinder the large-scale application of CA-based interventions in commercial aquaculture. Most studies to date have focused on a narrow range of species, primarily freshwater fish such as carp, tilapia, and trout, and have largely overlooked economically significant crustaceans and marine finfish. In addition, the majority of experimental work has been conducted under controlled laboratory or pilot-scale conditions. As a result, the efficacy, optimal dosing, and practical outcomes of CA supplementation in full-scale pond or cage farming systems remain poorly understood. Furthermore, there is limited information on the long-term safety and environmental fate of CA and its derivatives in aquatic ecosystems, particularly regarding their impact on non-target organisms and sediment quality. Mechanistic understanding of how CA and CAPE influence gut microbiota, modulate host metabolic pathways, or regulate gene expression is also still in its early stages. These knowledge gaps underscore the need for further species-specific, mechanistic, and field-based research to fully realize the potential of caffeic acid and its derivatives in sustainable aquaculture.

## 4. Mechanisms of CA and Its Derivatives in Aquaculture

### 4.1. Antioxidant Properties

CA has a hydroxycinnamic acid structure, consisting of an aromatic ring with two hydroxyl groups and an unsaturated three-carbon side chain terminating in a carboxylic acid group. These structural features contribute to its strong antioxidant capacity by enabling effective free radical scavenging. Specifically, CA can delocalize unpaired electrons and donate hydrogen atoms, allowing it to function as a primary antioxidant that neutralizes reactive radicals [94,95]. The hydroxyl group in the para position further enhances radical stabilization. In addition to its primary antioxidant role, CA acts as a secondary antioxidant by chelating metal ions such as Fe^2+^ and Cu^2+^, which would otherwise catalyze the decomposition of peroxides into harmful radicals. However, the chelation of Cu^2+^ may sometimes result in its reduction to Cu^+^, triggering reactions that produce superoxide and hydroxyl radicals, thereby exhibiting pro-oxidant activity [96]. CA also inhibits the formation of ROS by targeting 5-lipoxygenase, an enzyme involved in converting arachidonic acid into leukotrienes, a process that contributes to ROS production [97]. Since excessive ROS levels can cause cellular damage, CA’s antioxidant activity plays a crucial role in mitigating these effects [98,99]. Moreover, CA interacts synergistically with endogenous antioxidants such as α-tocopherol (α-TOH) and ascorbic acid in fish muscle tissue [81]. It protects α-TOH in tissue membranes and is regenerated by ascorbic acid, thereby enhancing the overall antioxidant defense system. Notably, CA also inhibits lipid oxidation in marine species such as mackerel (*Scomber scombrus*) by suppressing lipoxygenase activity at concentrations ranging from 10 to 100 μM [100].

Dietary supplementation with CA has demonstrated significant antioxidant benefits in various aquaculture species. For instance, in Nile tilapia, diets supplemented with 5 g/kg of CA significantly enhanced the activity of key antioxidant enzymes, including SOD, CAT, and GPx [22]. In rainbow trout gill cells exposed to SNP-induced oxidative stress, CA treatment reduced LDH leakage, prevented DNA fragmentation, and inhibited caspase-3 activation, thereby protecting cells from oxidative damage and apoptosis [26]. This protective effect was further supported by the upregulation of antioxidant-related genes, including *GST* and *MTs*. In beluga sturgeon, dietary supplementation with CA (5–10 g/kg) over 56 days significantly enhanced the activities of *CAT*, *SOD*, and *GST* while also improving growth performance and overall health [20]. Similarly, in common carp, supplementation with CA (5 g/kg feed) in combination with *B. coagulans* (2 × 10^7^ CFU/g feed) significantly increased antioxidant enzyme activities and gene expression, effectively improving oxidative stress management and leading to higher survival rates under pathogenic challenges [21]. Thus, integrating CA into aquafeeds could significantly contribute to sustainable aquaculture practices by reducing reliance on synthetic antioxidants and enhancing fish health and productivity under intensive rearing conditions.

### 4.2. Pro-Inflammatory and Anti-Inflammatory Properties

In aquaculture, fish are routinely subjected to environmental stressors, including abiotic factors such as temperature fluctuations and pollutants, and biotic factors such as pathogens, which induce oxidative stress and inflammatory diseases [101,102,103]. PRRs, including TLRs, NLRs, and RLRs, detect PAMPs and DAMPs, initiating immune responses [104,105]. These PRRs activate signaling cascades, such as the MyD88-dependent pathway, leading to the activation of transcription factors like NF-κB, which regulate pro-inflammatory cytokines, including interleukin-1 beta (IL)*-1β*), *IL-6*, and *TNF-α*, essential for pathogen defense [106]. However, prolonged or excessive activation of TLR signaling under chronic stress can disrupt immune homeostasis and lead to inflammatory disorders [107].

CA exhibits both pro-inflammatory and anti-inflammatory effects in a context-dependent manner. It may initially stimulate a pro-inflammatory response to enhance host defense, as evidenced by the upregulation of cytokines such as *IL-1β*, *TNF-α*, and *TLR7*. Subsequently, CA contributes to the resolution of inflammation by inhibiting *NF-κB* activation through suppression of *IκB* kinase phosphorylation and proteasomal degradation, thereby helping to restore immune homeostasis and prevent tissue damage [108].

The dual inflammation-regulatory properties of CA have been demonstrated in various aquaculture species. In Nile tilapia, CA-enriched diets significantly upregulated the expression of pro-inflammatory cytokines, including *IL-1β*, *TNF-α*, and *TLR7*, indicating a robust early immune response to infection [22]. Additionally, CA enhanced innate immunity by increasing phagocytic activity, respiratory burst, and serum myeloperoxidase activity. In common carp, the combination of CA and *B. coagulans* further amplified the expression of inflammatory cytokines such as *IL-1β*, *IL-8*, and *TNF-α* in liver tissues while increasing serum levels of total immunoglobulin, lysozyme activity, and ACH_50_ [21]. In rainbow trout, CA treatment prevented inflammation-induced cellular damage by reducing oxidative stress and inhibiting apoptotic pathways [26]. Similarly, dietary supplementation with CA in beluga sturgeon improved immune parameters, including total protein, immunoglobulin, and lysozyme activity, while upregulating immune-related genes such as *IL-3 NFI-3*, thereby contributing to an anti-inflammatory effect [20].

Although research on the mechanisms of action of immunostimulants in aquatic species remains relatively limited, CA and its derivatives have garnered considerable attention for their potential as effective immunomodulatory agents in aquaculture. Dietary supplementation with CA at a concentration of 5 g/kg has been shown to modulate both innate and adaptive immune responses, enhance antioxidant capacity, and improve survival rates during infections caused by *A. veronii* in Nile tilapia [22]. Similarly, in beluga sturgeon, supplementation with CA at concentrations of 5–10 g/kg has been associated with improvements in growth performance, digestive enzyme activity, and serum immune parameters [20]. In common carp, a diet enriched with CA and *B. coagulans* (2 × 10^7^ CFU/g feed) improved growth, immune parameters, and survival rates during *Aeromonas hydrophila* infection compared to untreated controls [21]. These findings support CA’s potential as sustainable alternatives to antibiotics in aquaculture by enhancing host defense while regulating inflammation, though careful dosage optimization is essential to avoid immunosuppression or metabolic stress.

### 4.3. Digestive System

CA and its derivatives are well-studied plant-derived phenolics known to enhance digestive physiology in various aquaculture species. Several studies have demonstrated that dietary supplementation with CA improves growth performance by stimulating digestive enzyme activity. For instance, in beluga sturgeon, dietary CA at 5–10 g/kg significantly enhanced growth and elevated the activities of key digestive enzymes, including amylase, lipase, and pepsin, compared to untreated controls. Similarly, supplementation with chlorogenic acid (CGA) at 600–800 mg/kg improved growth and increased digestive enzyme activities in rainbow trout [45]. In crucian carp (*C. auratus*), dietary CGA at 200 mg/kg significantly elevated intestinal amylase and lipase activities, which was accompanied by improved weight gain [37].

Comparable effects have been reported in other aquaculture species, including common carp [36], grass carp [39], loach (*Misgurnus anguillicaudatus*) [42], yellow pond turtle (*Mauremys mutica*) [48], Nile tilapia [58], Pacific white shrimp (*Litopenaeus vannamei*) [60], and yellow croaker (*Larimichthys polyactis*) [65]. These findings collectively suggest that CA and its derivatives play a beneficial role in promoting digestive enzyme activity, thereby enhancing nutrient utilization and supporting growth in a broad range of aquatic species.

**Figure 2:**
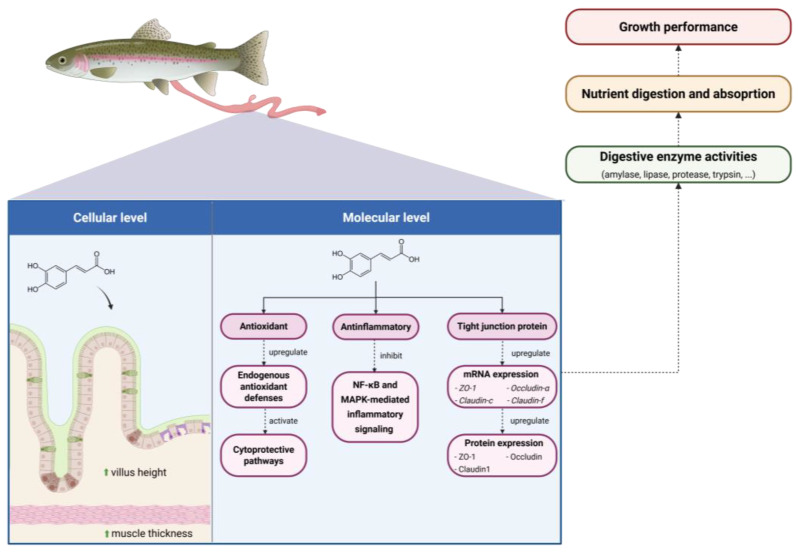
The mechanism of caffeic acid and its derivatives on digestive enzymes of aquatic species.

Mechanistically, the digestive benefits of CA and its derivatives are likely attributed to their potent antioxidant and anti-inflammatory properties. CA compounds enhance endogenous antioxidant defenses by upregulating enzymes such as SOD, CAT, and GPx, and by activating cytoprotective signaling pathways such as Nrf2. Concurrently, they inhibit inflammation by suppressing NF-κB- and MAPK-mediated signaling pathways (Figure 2). In Nile tilapia, dietary CA supplementation improved intestinal morphology, including increased villus height and muscle thickness in both the foregut and hindgut regions [23]. In Amur ide, supplementation with 0.04% CGA significantly upregulated the expression of tight junction-related genes and proteins, including ZO-1, occludin-α, claudin-c, and claudin-f mRNA, as well as ZO-1, occludin, and claudin-1 proteins [33], suggesting enhanced intestinal barrier integrity. Additionally, CAPE administration in zebrafish reduced the expression of pro-inflammatory cytokines *TNF-α* and *IL-1β*, while increasing the anti-inflammatory cytokine *IL-10*, further implying the suppression of *NF-κB* and promotion of anti-inflammatory signaling [24].

### 4.4. Immunological Properties

Multiple studies have demonstrated that dietary CA functions as a potent immunostimulant in aquaculture species. In Nile tilapia, for instance, supplementation with 5 g/kg CA for 60 days significantly enhanced innate immune parameters, including increased phagocytic and respiratory burst activities, elevated serum lysozyme and MPO activities, and upregulation of immune-related genes (IL-1β, TNF-α, IL-8, IFN-γ, and IgM) in liver tissue [22]. Similarly, beluga sturgeon fed 5–10 g/kg CA exhibited significantly higher serum lysozyme activity, total immunoglobulin, and total protein levels compared to controls [20]. In rainbow trout and several other species, CGA has been shown to enhance nonspecific immune responses, such as complement activity and lysozyme levels, and improve resistance to pathogenic infections [17,33,34,40,42,43,45]. Collectively, these findings indicate that CA and its derivatives stimulate both the cellular and humoral components of fish immunity.

**Figure 3:**
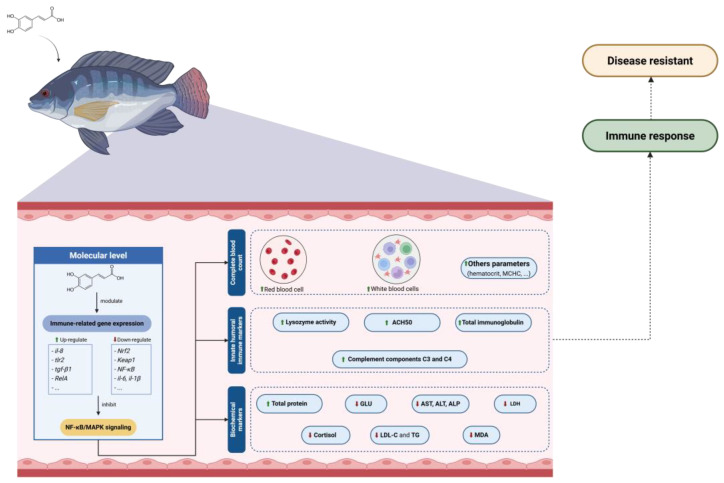
The mechanism of caffeic acid and its derivatives on immune systems on aquatic species.

In addition to immune enhancement, CA compounds exert anti-inflammatory effects that help maintain immune balance (Figure 3). In various aquaculture-relevant models, CA supplementation has been shown to reduce pro-inflammatory cytokine expression and suppress NF-κB signaling. For example, CAPE administration in zebrafish downregulated TNF-α and IL-1β, while upregulating anti-inflammatory IL-10 expression [109]. By mitigating oxidative stress and inflammation, CA derivatives help prevent immune overactivation and tissue damage, while preserving effective pathogen defense.

Consistent with these mechanisms, numerous studies report improved disease resistance following dietary CA supplementation. In Nile tilapia, feeding 5 g/kg CA resulted in survival rates against *A. veronii* comparable to those observed in antibiotic-treated groups [22]. Similarly, rainbow trout supplemented with CGA exhibited enhanced survival following *Y. ruckeri* challenge [50], and Pacific white shrimp infected with *Vibrio parahaemolyticus* also showed improved survival after CGA treatment [43]. These protective effects reflect both strengthened innate immunity and reduced pathological inflammation. Furthermore, CA acts as a cellular antioxidant, enhancing the activities of SOD, CAT, and GPx, while reducing ROS and MDA levels, thereby preserving immune cell function and integrity.

In summary, CA and its derivatives serve as potent immunomodulators in fish, enhancing innate responses (e.g., lysozyme, respiratory burst), promoting adaptive markers (e.g., *IgM*), suppressing pro-inflammatory signaling (e.g., *NF-κB*, *TNF-α*, and *IL-6*), and upregulating anti-inflammatory and antioxidant defenses. These complementary actions collectively explain the consistent improvements in immune status and disease resistance observed across diverse aquaculture species fed CA-enriched diets.

### 4.5. Using as an Alternative to Antibiotics

The antimicrobial properties of CA (Table 3) have been attributed to its ability to disrupt bacterial metabolism and compromise cell membrane integrity [110,111,112]. Specifically, CA penetrates the semi-permeable bacterial membrane, decomposes in the cytoplasm, and neutralizes intracellular pH [113]. This acidification impairs critical metabolic processes, such as enzymatic activity, and disrupts key cellular enzymes, ultimately leading to bacterial cell death [114,115]. These mechanisms are effective against both Gram-positive and Gram-negative bacteria. At 1.0 mg/mL, CA inhibits the growth of *Staphylococcus aureus*, a Gram-positive pathogen associated with biofilm formation and antibiotic resistance [111], likely through suppression of proline dehydrogenase (PRODH), a key enzyme in energy production and redox regulation [116]. At 10 mg/mL, CA further suppresses the secretion of α-hemolysin, a virulence factor responsible for erythrocyte hemolysis [112]. In antibiotic-resistant *S. aureus* strains (RN-4220 and-1199B), CA (at 1 mg/mL) inhibited the efflux pumps MrsA and NorA, which are key contributors to antibiotic resistance [117]. Furthermore, in silico studies have shown that CA effectively inhibits the efflux pumps tetR and tetM, critical for tetracycline resistance, underscoring its potential to combat efflux-mediated antibiotic resistance [118]. Beyond *S. aureus*, CA demonstrates broad-spectrum antibacterial activity, with efficacy confirmed against *Staphylococcus epidermidis* and *Klebsiella pneumoniae* at a concentration of 5 mg/mL [119]. Additionally, CA has been shown to inhibit the growth of *Escherichia coli*, *Pseudomonas aeruginosa*, *Listeria monocytogenes*, and *S. aureus* at 1 mg/mL [120]. Remarkably, these inhibitory effects were observed both independently and in combination with antibiotics such as gentamicin, ciprofloxacin, and streptomycin, highlighting the potential synergistic applications of CA in combating bacterial infections [120]. In vitro studies have demonstrated the considerable potential of CA in controlling aquaculture pathogens, where CA exhibits inhibitory activity against *Aeromonas* spp., with minimum inhibitory concentrations ranging from 1.56 to 3.12 mg/mL [121]. The antimicrobial activity of CA has also been shown to be pH-dependent, with concentrations as low as 0.4% (*w/w*) effectively inhibiting microbial growth within a pH range of 5 to 7 [122,123].

The combination of CA with other treatments has been investigated as a strategy to enhance its antimicrobial efficacy. For instance, CA combined with UV-A light effectively inactivated foodborne pathogens such as *E. coli*, *Salmonella enterica* serovar *Typhimurium*, and *L. monocytogenes* by causing significant damage to bacterial cell membranes, intracellular structures, and enzymatic activity, ultimately resulting in bacterial death [152]. In aquaculture, CA supplementation has been associated with beneficial alterations in the intestinal microbiota, which positively influence growth performance, nutrient utilization, immune responses, and pathogen resistance [115]. CA’s broad-spectrum antibacterial activity has also been demonstrated against major aquaculture pathogens, including *Y. ruckeri*, *Listonella anguillarum*, *Streptococcus iniae*, *Edwardsiella tarda*, and *Citrobacter* spp., with studies confirming its efficacy against all five bacteria [82]. Furthermore, the development of CA derivatives, such as CAPE, has enhanced its stability and antimicrobial activity, particularly through inhibitory effects on bacterial efflux pumps, which are often responsible for antibiotic resistance [153]. Additionally, CA and its derivatives have shown promise in in silico screenings for antiviral applications, underscoring their versatility in combating a wide range of pathogens [154,155]. Despite these promising trends, most research on CA compounds remain limited to planktonic microbial cultures or cell-based viral assays, which fail to capture the complex host–pathogen–environment interactions characteristic of aquaculture systems. Consequently, there is a notable lack of in vivo data evaluating the efficacy of CA and its derivatives against common aquatic pathogens such as *Vibrio* spp., *Aeromonas* spp., and viral agents affecting fish and shrimp. Furthermore, key factors such as the stability, bioavailability, and potential off-target effects of these phenolics in saline or brackish water environments have not been systematically investigated. These gaps significantly hinder the translation of laboratory findings into field-relevant aquaculture applications.

## 5. Challenges and Limitations

Despite its promising immunostimulant, antioxidant, and antimicrobial properties, the application of CA in aquaculture presents several critical challenges and limitations. A primary concern is its dose-dependent effect: while optimal concentrations (5–10 g/kg) yield beneficial outcomes, excessive doses can induce adverse effects, including pro-oxidant activity and metabolic stress. For example, CA-mediated chelation of Cu^2+^ ions reduces them to Cu^+^, generating ROS such as superoxide and hydroxyl radicals, thereby exacerbating oxidative stress rather than mitigating it [96]. These dynamics underscore the need for precise dose optimization and conservative, stepwise titration from the lower bound of effective ranges, accompanied by routine monitoring of oxidative-stress biomarkers (SOD, CAT, GPx, and MDA), FI, CF, and behavior during pilot use. Given the predominance of short- to medium-term trials, long-term endpoints, metabolic burden, tissue residues, environmental fate, microbiome stability, and durability of effect should be prioritized. Although phenolic additives likely impose lower selective pressure than antibiotics, adaptive tolerance cannot be excluded; stewardship should therefore favor minimum effective dosing with periodic re-evaluation.

**Figure 4:**
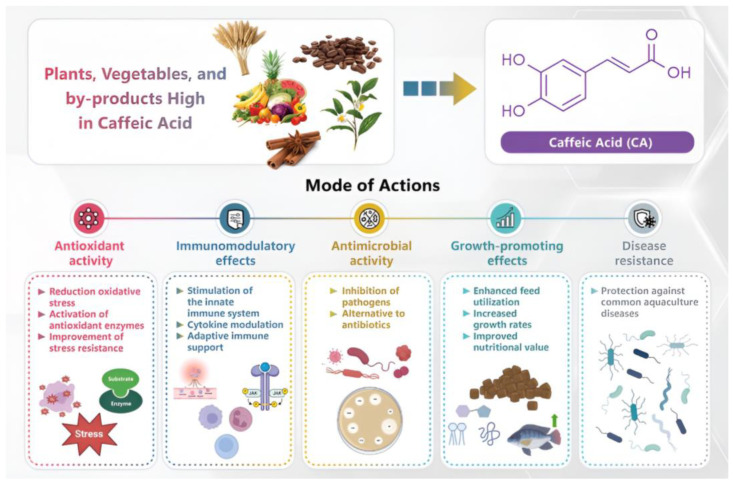
The key biological roles of caffeic acid, a phenolic compound derived from different sources.

**Figure 5:**
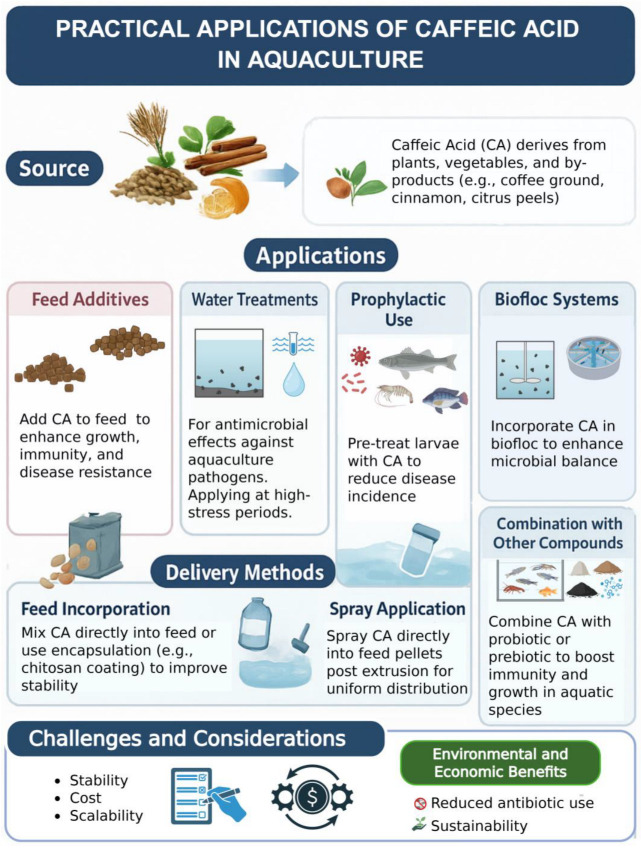
Practical applications of caffeic acid in aquaculture, including delivery methods, challenges, and key considerations.

Another limitation is the bioavailability and stability of CA in aquatic environments and fish metabolism. CA degrades under environmental factors such as pH fluctuations, light exposure, and enzymatic activity, significantly reducing its efficacy in dynamic aquaculture settings [122,123,156]. Its antimicrobial activity is pH-dependent, with optimal performance between pH 5 and 7, limiting its applicability in less controlled environments [156]. Additionally, the metabolic pathways and excretion mechanisms of CA in aquatic species remain poorly characterized. Rapid metabolization may limit tissue retention, reducing its prolonged protective effects against pathogens and oxidative stress. Further research is essential to elucidate CA’s long-term physiological impacts and ensure its sustainable use in aquaculture (Figure 4 and Figure 5).

The synergistic interactions between CA and other dietary components or probiotics, such as *B. coagulans*, show promise for enhancing fish health [21]. However, these combinations introduce complexities, including the need to optimize formulations, prevent antagonistic interactions, and ensure feed stability. CA’s efficacy varies by species, influenced by metabolic rates, diet composition, and environmental stressors. Although CA exhibits potent antimicrobial effects against pathogens like *Aeromonas* spp. [121], its mechanism of action in aquatic pathogens remains inadequately defined. CA disrupts bacterial metabolism and cell membrane integrity, but its long-term impact on gut microbiota and its potential for fostering microbial resistance in aquaculture systems are not fully understood. Overreliance on CA as an antibiotic alternative could inadvertently promote resistance, mirroring challenges faced with synthetic antimicrobials [120,153].

Cost-efficiency and scalability remain significant barriers to the commercial viability of CA in aquaculture. The industrial-scale production of purified CA or its derivatives is considerably more expensive than conventional immunostimulants or antibiotics. While plant-based extraction offers a sustainable alternative, the resource-intensive purification processes required to achieve consistent quality and bioactivity limit its practicality, particularly for small-scale aquaculture operations.

Despite these challenges, CA may still be economically justifiable in high-value species or during critical production stages such as larval rearing and broodstock management, where improved health and survival outcomes can offset the higher feed costs. Moreover, several cost-reduction strategies hold promises for broader application. These include the use of CA-rich agricultural byproducts (e.g., coffee pulp, cascara, and fruit skins), encapsulation technologies to enhance bioavailability and reduce effective dosages, and advances in biotechnological production that may lower manufacturing costs. Collectively, these approaches could enhance the cost-effectiveness and scalability of CA for use in sustainable aquafeed formulations.

## 6. Future Perspective and Research Directions

The growing interest in CA and its derivatives as a natural alternative to enhance fish health and productivity in aquaculture presents significant opportunities for future research. However, fully realizing CA’s potential requires addressing key challenges, including formulation optimization and a comprehensive understanding of its biological mechanisms. A critical research priority is developing advanced delivery systems to improve CA’s bioavailability and stability in aquaculture environments. As a phenolic compound, CA is highly susceptible to degradation by light, oxygen, and pH fluctuations, limiting its effectiveness in water-based systems [122]. Encapsulation techniques such as nano-emulsions, liposomes, and polymeric nanoparticles should be explored to protect CA from environmental degradation and enhance its gastrointestinal absorption in fish. Terrestrial animal studies show that nanoencapsulation significantly improves polyphenol stability and bioavailability [157,158,159,160], suggesting similar approaches could be adapted for aquaculture. Microencapsulation could also enable targeted delivery to specific tissues, maximizing therapeutic benefits while minimizing required dosages [161,162]. To guide implementation, priority steps are stability and bioavailability benchmarking of free versus encapsulated CA across pelleting, storage, and immersion, and harmonized dose–response trials with shared endpoints to link delivery strategies to mechanisms and performance. Additionally, the applications of CA to diverse aquaculture species also requires deeper investigation into species-specific responses. While promising results have been observed in various fish species, differences in physiology, metabolism, and immune responses may affect CA’s efficacy. Comparative studies across species, including crustaceans and mollusks, are essential to evaluate CA’s immunostimulatory, antioxidant, and antimicrobial effects under diverse husbandry conditions. Elucidating the metabolic pathways through which CA interacts with host tissues will provide critical insights into its long-term physiological impacts and tissue residues, ensuring safe and effective aquaculture use [163,164]. Synergistic combinations of CA with probiotics, prebiotics, and other functional additives represent another promising research avenue. Compared with probiotics, CA and related phenolic acids are non-viable and thermally robust, simplifying storage and pelleting but not conferring colonization or barrier functions. Relative to essential oils, CA compounds are less volatile and easier to dose consistently, with fewer aroma-linked palatability constraints. Probiotics provide enzymatic and mucosal benefits, essential oils offer broad antimicrobial actions, and CA compounds most consistently deliver antioxidant, immunomodulatory, and anti-inflammatory effects. These profiles are complementary; accordingly, factorial trials are warranted to quantify additivity or synergy in growth performance and disease resistance. Such combinations could produce multifunctional aquafeeds that enhance fish health, growth, and disease resistance. Understanding CA’s interactions with gut microbiota and its influence on host immunity and metabolism through metagenomics and transcriptomics is crucial. Further research into CA’s antimicrobial mechanisms against aquaculture pathogens is needed to establish its potential as an antibiotic alternative. Proteomics and metabolomics could identify CA’s cellular targets in both Gram-positive and Gram-negative bacteria. Additionally, investigating the potential for antimicrobial resistance under prolonged CA exposure is essential for sustainable use in aquaculture systems.

Research into the genetic and epigenetic regulation of CA’s effects in fish is another valuable direction. Limited knowledge exists regarding CA-induced epigenetic modifications, such as DNA methylation and histone acetylation, which may regulate immune responses and oxidative stress pathways, providing insights into long-term impacts on fish health and resilience to environmental stressors. Economic feasibility and sustainability studies are critical for CA’s commercial adoption. Although CA can be extracted from plant sources such as coffee, fruits, and herbs, large-scale production requires cost-effective and environmentally sustainable methods. Research into sustainable sourcing and cost-reduction strategies, such as plant waste valorization and synthetic biology approaches, will be critical to improving CA’s economic feasibility and supporting its broader adoption in commercial aquaculture. Future research should explore alternative plant sources or employ synthetic biology for industrial-scale production. Life cycle assessments of CA-based feeds could further evaluate their environmental impact, supporting the transition to sustainable aquaculture practices.

While this review focuses on the application of CA in aquaculture, it is important to acknowledge that CA has also demonstrated a wide range of health benefits in mammalian systems, including antioxidant, anti-inflammatory, and metabolic regulatory effects [14,15]. Although these findings are beyond the primary scope of the current review, they may offer valuable insights for future cross-species comparisons. Of particular note are CA’s reported anti-obesity effects in mammals, which are linked to its ability to regulate lipid metabolism and reduce excessive fat accumulation, mechanisms relevant to metabolic disease prevention [165,166]. However, in aquaculture, the goal is not to increase fat deposition but to enhance feed efficiency, promote lean muscle growth, and support overall metabolic health. Empirical studies in fish have consistently shown that CA supplementation improves growth performance, antioxidant capacity, and immune responses without negatively affecting body weight or condition. Future studies should further investigate CA’s effects on lipid deposition, body composition, and energy allocation in aquaculture species to clarify its long-term nutritional impact and optimize its use in functional feed formulations.

## 7. Conclusions

CA has emerged as a promising natural feed additive in aquaculture, with increasing evidence supporting its ability to enhance growth performance, stimulate digestive enzyme activity, and modulate immune responses across various fish species. However, its efficacy is influenced by species-specific physiological responses, optimal dosing strategies, and the stability of its delivery within feed systems. To facilitate the effective integration of CA into aquaculture practices, future research should prioritize the elucidation of its underlying mechanisms of action, bioavailability, and long-term impacts on fish health and microbial resistance. Additionally, the development of scalable, cost-effective production and formulation methods is essential to support its widespread adoption. Addressing these research gaps will reduce reliance on antibiotics and advance the sustainability of aquaculture systems.

## Figures and Tables

**Table 1:** Summary of caffeic acid and its derivatives effects in aquaculture species.

Compound	Species	Supplementation	Observed Effects	References
Caffeic acid	Atlantic horse mackerel (*Trauchurus trauchurus*)	10 to 200 ppm (*w*/*w*, 0.001–0.020%)	- High efficacy in inhibiting lipid oxidation for fish muscle due to its capacity for donating 12.2 µmol electrons/mg antioxidant	[19]
	Beluga sturgeon (*Huso huso*)	5–10 g/kg CA over 56 days	- Increased antioxidant enzyme activities (SOD, CAT, and GST). - Improved growth performance and immune parameters	[20]
	Common carp (*Cyprinus carpio*)	5 g/kg CA + *Bacillus coagulans* (2 × 10^7^ CFU/g feed)	- Enhanced oxidative stress management - Upregulated immune-related gene expression - Higher survival rates under pathogenic challenges	[21]
	Nile tilapia (*Oreochromis niloticus*)	5 g/kg CA in diet	- Enhanced activity of antioxidant enzymes (SOD, CAT, and GPx) - Improved immune responses - Increased survival rates against infections	[22]
		0.52 mmol/kg CA	- Increased FW, WG, ALB, TP, ALB, and BUN - Decreased FCR, AST, GLU, TG, and LDH - Improved hardness, gumminess, adhesiveness, and resilience of muscle - Increased villus height and muscle thickness of the foregut and hindgut	[23]
	Rainbow trout (*Oncorhynchus mykiss*) adipocyte cell	Incubated with vehicle + 50 μM CA	- Increased adipocyte viability without affecting proliferation - CA in combination with rosiglitazone significantly reduced the enhanced *PPARγ* protein expression signal produced by rosiglitazone alone - Lower level of lipid storage in the cell - Increased lipoprotein lipase expression in liver - Decreased triacylglycerol levels in fasting	[24]
	Sea bass (*Lateolabrax japonicas*)	Dipping fresh sea bass fillets in 2.0 g/L CA, ultrasonic + 2.0 g/L CA	- Slowed the growth of total viable count - Improved the water-holding capacity of fish fillets - Inhibited lipid oxidation in fish fillets - Maintained the better quality and extended the shelf-life of sea bass by at least four days	[25]
	Zebrafish larval (*Danio rerio*)	Exposure in 50 μM CA	- Decreased larval adiposity and adiposity in the head	[24]
	Rainbow trout (*Oncorhynchus mykiss*)	CA treatment in gill cells exposed to oxidative stress	- Reduced DNA fragmentation, prevented caspase-3 activation - Protected cells from oxidative damage and apoptosis	[26]
Caffeic acid phenethyl ester	Grass carp (*Ctenopharyngodon idellus)*	0, 200, 500, and 800 mg/kg in high-fat diet	- Reduced the HFD-induced increase in feed conversion ratio - Promoted adipocyte hyperplasia, leading to healthy adipose tissue remodeling - Decreased immune cell infiltration, promoted the ability of adipose tissue to handle fat, reduced lipid accumulation - Alleviated hepatopancreas steatosis by reducing hepatopancreas lipid absorption and synthesis - Decreased the expression of pro-inflammatory factors in adipose tissue, hepatopancreas and muscle - Decreased levels of serum LD, ALT, AST, and LDLC	[27]
		0, 200, 500, and 800 mg/kg in high-carbohydrate diet	- Reduced the adverse effects of a high-carbohydrate diet - Peroxisome proliferator-activated receptor γ was activated by CAPE in adipose tissue - Promoted the lipolysis and fatty acid β-oxidation in the adipose tissue, hepatopancreas, and muscle - Improved glucose uptake and utilization-related gene expression in the hepatopancreas and muscle - Alleviated hepatic steatosis, and promoted mammalian target of rapamycin gene expression in muscle - Inhibited inflammatory response in the adipose tissue, hepatopancreas, and muscle - Reduced the levels of ALT, AST, GLU, LD, LDLC, and TG	[28]
	Five-day post-fertilization zebrafish (*Danio rerio*) larvae	Exposed to 125 μM neomycin and CAPE (50, 100, 250, 500, or 1000 μM) in 1 h	- Decreased neomycin-induced hair cell loss in the neuromasts, apoptosis, and mitochondrial damage - Structures of mitochondria and hair cells were preserved when exposed to 125 μM neomycin and 500 μM CAPE	[29]
Caffeic acid complexed with Cr(III) and Pb(II)	Common carp (*Cyprinus carpio*), water flea *(Daphnia magna*), green algae (*Selenastrum capricornutum*)	Exposure	- No toxicity in the studied aquatic organisms (CL_50_/CE_50_ > 100 mg/L) - Flotation effluents containing Pb(II) and Cr(III), obtained after ion and precipitate flotation processes, are not harmful for common carp - Removal of toxic metals (Pb and Cr) in flotation processes	[30]
Carbodiimide-mediated grafting of caffeic acid on chitosan	Pompano (*Trachinotus ovatus*)	Immersed fresh fillet with 1% CS and CS-g-CA solution	- Lowered the softening rate - Increased the quality of fish fillet - Inhibited the deterioration of texture	[31]
Chitosan-grafted caffeic acid	Pompano (*Trachinotus ovatus*)	Fresh fish slices were treated with ultrasonic, CS-g-CA, and ultrasonic + CS-g-CA	- Increased the surface hydrophobicity and the total sulfhydryl content - Maintained the secondary and tertiary structure of myofibrillar proteins - Inhibited protein degradation - Protected the myofibril microstructure by maintaining the compact arrangement of muscle fibers - Improved the sensory properties	[32]
Chlorogenic acid	Amur ide (*Leuciscus waleckii*)	0.04%	- Alleviated LPS-induced intestinal barrier dysfunction (decreasing the levels of 5-HT, D-LA, ET-1 and DAO in serum, increasing ZO-1, occludin-α, claudin-c, claudin-f mRNA, ZO-1, occludin, and claudin-1 protein expression) - Improved intestinal morphology - Upregulated TGF-β and Bcl-2 mRNA expression - Downregulated NF-κBp65, TNF-α, Bax, caspase-3, caspase-9 mRNA, NF-κBp65, Bax, and caspase-3 protein expression - Reversed LPS-induced reduction in GSH-PX, CAT, T-SOD and T-AOC in intestines - Alleviated LPS-induced decrease in Nrf2, HO-1, CAT, SOD mRNA and Nrf2 protein expression, and increase in Keap1 mRNA	[33]
	Asian swamp eel (*Monopterus albus*)	250, 500, 750 mg/kg	- Increased WGR, Hb, and HDL - Decreased TG, GLU, LDL, GSP, and MDA levels - Increased GPx, CAT, SOD, LYZ, and ALP levels	[34]
	Blackspotted croaker (*Protonibea diacanthus*)	0, 100, 200, 400, 800, 1600 mg/kg	- Reduced muscle oxidative stress and inflammation and muscle fiber content. - Increased muscle toughness (hardness, chewiness, shear force, and gumminess), collagen content and collagen deposition in muscle	[35]
	Common carp (*Cyprinus carpio*)	10^7^ CFU/g *Lactobacillus helveticus*, 550 mg/kg CGA, and a combination of both elements	- Increased growth parameters, digestive enzyme activities (trypsin, chymotrypsin, pepsin, protease), total protein, lysozyme activity, ACH50, and total immunoglobulin - Decreased MDA, AST, ALT, ALP, GLU, and cortisol levels	[36]
	Crucian carp (*Carassius auratus*)	200 mg/kg	- Increased growth performance (FW, WG, and SGR), digestive enzyme activity, TG, non-specific immune enzyme activities of intestinal, and liver tissues - Improved antioxidant capacity of intestinal, muscle, and liver tissues - Decreased FCR - Upregulated the expression of lipid metabolism-related genes in the liver - Enhanced the relative abundance of intestinal microbes, *Fusobacteria* and *Firmicutes* - Degraded the relative abundance of *Proteobacteria*	[37]
		100, 200, 400, 800 mg/kg	- Increased WGR, SGR, FER, PER; serum levels of LZM; AKP activity; C3 and C4 concentration; SOD, CAT, and GsH-Px activities; the mucosal fold height - Decreased MDA content, DAO, D-LA, and ET-1 in intestines - Upregulated IL-10, TGF-β, Nrf2, HO-1, SOD, CAT, and GPX expression - Downregulated NF-κB, TNF-α, and IL-1β expression	[38]
	Grass carp (*Ctenopharyngodon idellus*)	CGA (400 mg/kg), quercetin (QC, 400 mg/kg), and their combinations	- Increased WG, digestibility of dry matter, protein, digestive enzyme (amylase, protease), activities of SOD and GSH-Px - Decreased FCR and MDA level - Increased flesh hardness, water-holding capacity, total collagen, heat-insoluble collagen and n-3 polyunsaturated fatty acids contents - Reduced mesenteric lipid-somatic index, flesh n-3/n-6 ratio, serum triglycerides, lipid content in flesh	[39]
	Koi carp (*Cyprinus carpio*)	200, 400, 600, 800 mg/kg	- Increased body color indices (redness, yellowness, and lightness) - Increased lysozyme, SOD, CAT, GSH and GSH-Px activities - Upregulated TGF-β expression in the head-kidney	[40]
	Largemouth bass (*Micropterus salmoides*)	60, 120, 180, and 240 mg/kg	- Increased FBW, SGR, WGR, and GSH levels - Decreased FCR, TC and TG levels, MDA content, CAT, SOD, and GPx activities - Reduced mRNA levels of SOD, CAT, Nrf2, Keap1, and NF-κB - Elevated GPx, IL-8, TLR2, and RelA expression in the liver	[17]
		300 and 600 mg/kg in high-fat diet	- Regulated expression of gene responsible for lipid metabolism, inflammation (HSL, ATGL, APOA1, IL-8, and TNF-α), and antioxidant enzymes (CAT, SOD, and GPx) - Decreased TG and TC in plasma; MDA and NEFA in liver	[41]
	Loach (*Misgurnus anguillicaudatus*)	200, 400, 600 and 800 mg/kg	- Increased FBW, WGR, and SGE; enzyme activities in liver and intestine (protease, lipase, and amylase); T-AOC, CAT, SOD, GSH, GSH-Px, AST, and ALT in liver; C3 and C4 in serum; IgM; LYZ - Decreased MDA level	[42]
	Pacific white shrimp (*Litopenaeus vannamei*)	CGA (200 mg/kg), low-dose drug combination (100 mg/kg CGA + 7.5 mg/kg FFC), moderate-dose drug combination (200 mg/kg CGA + 15 mg/kg FFC), and a high-dose drug combination (400 mg/kg CGA + 30 mg/kg FFC)	- Decreased cumulative mortality over 5 days after infection with *V. parahaemolyticus* - Increased the immune parameters - Improved the hepatopancreatic tubule structure and integrity	[43]
		100, 200 and 400 mg/kg	- Increased growth performance and the activities of TAS, SOD, GSH-Px, and CAT in hepatopancreas - Improved the resistance against the combined stress of low salinity and nitrite	[44]
	Rainbow trout (*Oncorhynchus mykiss*)	200, 400, 800 mg/kg	- Increased FW, WG, SGR; digestive enzyme activities (protease, amylase); and activities of NBT, NPO, LYZ, Ig, CAT, C3, C4, TP, AST, ALP, ALT, LDH, TP, and GLO - Decreased FCR, MDA - Enhanced resistance against *Y. ruckeri*	[45]
	Red swamp crayfish (*Procambarus clarkii*)	Injected 50 mg/kg	- Regulated innate immune defense to reduce viral gene transcription - Induced apoptosis to restrict viral spread - Enhanced anti-inflammatory and antioxidant activities to alleviate oxidative and inflammatory damage caused by WSSV	[46]
	Spotted sea bass (*Lateolabrax maculatus*)	100, 200, 300, and 400 mg/kg CGA in high-fat diet	- Decreased the HFD-induced hyperlipemia - Reduced serum AST, ALT, and MDA levels - Improved liver T-OAC activity - Hepatocytes were arranged more neatly, reduction in lipid deposition and hemolysis in the liver - Increased mucosal thickness, as well as villus number and width - Increased the intestinal *Bacteroidetes* to *Firmicutes* ratio and decreased the abundance of *Vibrio*	[47]
	Yellow pond turtles (*Mauremys mutica*)	100, 200, 400, 800 mg/kg	- Enhanced FW, WGR, and SGR - Reduced FCR - Improved intestinal digestive enzyme activities (amylase, lipase, and trypsin) - Promoted intestinal morphological development, and increased villus length and density. - Increased AKP, ACP, complement C3, complement C4, SOD, and CAT activities - Decreased MDA level - Modulated liver gene expression (downregulated MHC I and MHC II, enriched KEGG pathways related to Th1 and Th2 cell differentiation, Th17 cell differentiation, asthma, and other immune-inflammatory conditions) - Increased the relative abundance of *Cetobacterium* and reduced the abundance of *Escherichia-Shigella* and *Treponema* in the intestinal microbiota.	[48]
Cinnamic acid	Rainbow trout (*Oncorhynchus mykiss*)	0.25, 0.50, 0.75, 1.50	- Decreased intestinal and stomach pH, serum triglyceride, and AST, ALT, LDH and ALP levels - Increased the serum SOD and liver SOD2, CAT, GST, GPX1, GPX4 and GR gene expression responses	[49]
		Mix of *Bacillus subtilis* and trans-cinnamic acid (25 mg/kg-25trcBS, 50 mg/kg-50trcBS, 75 mg/kg-75 trcBS, 150 mg/kg-150 trcBS)	- Increased granulocyte percentage, respiratory burst activity, phagocytic activity, phagocytic index, myeloperoxidase activity and total antiprotease activity - Improved disease resistance against *Y. ruckeri* (increasing survival rates and antibody titer)	[50]
Ferulic acid	Blunt snout bream (*Megalobrama amblycephala*)	100, 200 mg/kg	- Enhanced WGR, GI, PER, and CF - Reduced ALT, GLU, LDH, and MDA levels - Increased CAT, SOD, GPx, GRS, and GST activities - Downregulated the expression levels of inflammation-related genes (IL-6, IL-1β, and NF-κB), caspase-8 and endoplasmic reticulum stress-related genes (XBP1s, PERK, IRE1, CHOP, and BIP) - Activated ATF6α and ATF4 expression - Upregulated BCL-2 expression	[51]
	Carp (*Cyprinus carpio*)	200 mg/kg	- Normal brain tissue structure with tightly arranged neuronal fibers and increased number of neurons under AVM exposure - Increased the levels of acetylcholinesterase and tight junction proteins (ZO-1, occludin, and claudin11) in the carp brain under AVM exposure - Restored the imbalance of key oxidative and antioxidative parameters, reduced ROS accumulation in brain tissue, and ultimately alleviated the oxidative stress induced by chronic exposure to AVM - Modulated the Nrf2/Keap1 signaling pathway - Decreased the apoptotic rate - Downregulated transcription levels of Bax and caspase-3 - Upregulated the expression of Bcl-2, AgRp, NPY, and orexin in the hypothalamus	[52]
		400 mg/kg	- Alleviated gill tissue damage induced by chronic DFZ exposure - Downregulated IL-1β, IL-6 and TNF-α expression - Upregulated IL-10 and TGF-β1 expression - Reduced MDA levels and ROS accumulation - Increased CAT, GSH, and T-AOC activities - Suppressed the activation of the NF-κB signaling pathway and the NLRP3 inflammasome pathway in the gills	[53]
		400 mg/kg	- Increased serum complements C3 and IgM and activities of T-AOC and CAT in spleen under AVM exposure - Decreased MDA and GSH in spleen under AMV exposure - Downregulated TNF-α, IL-6, IL-1β, and iNOS mRNA levels under AVM exposure - Upregulated TGF-β mRNA levels under AVM exposure	[54]
		*Lactobacillus fermentum* (10^8^ CFU/g) and/or ferulic acid (100 mg/kg)	- Higher FW, WG, and SGRT - Lower FCR - Increased RBC, WBC, Hb, HCT; serum total protein and albumin levels; antioxidant enzymes (CAT, GPx, and SOD), and serum respiratory burst and lysozyme activity - Enhanced resistance against *Aeromonas hydrophila*	[55]
	Carp (*Cyprinus carpio* var. Jian)	0.10, 0.20, 0.30, 0.40 g/kg	- Improved FBW, WG, SGR, and FI - Increased FACs, caspase-3 and -8 activities, and activities of SOD and CAT in RBC - Decreased FDO, Na^+^, K^+^-ATPase, GPT, GOT and caspase-9 activities; GPx, O_2_^−^, OH, and MDA levels in the gills and RBC - Counteracted the Cu-induced reductions in Hct, RBC, HbC, and MCH	[56]
	Grass carp (*Ctenopharyngodon Idellus*)	50, 100, 200 mg/kg	- Improved growth performance and feed utilization - Reduced liver MDA, ATL, lipid deposition, TG, and TC - Increased GSA, SOD, bile acid of blood and liver, and HDLL - Upregulated mRNA expression of ZO-1, ZO-2, occludin, claudin-b, claudin-3, claudin-7a, claudin-12; FXR and CYP7A1	[57]
	Nile tilapia (*Oreochromis niloticus*)	Highly oxidized fish oil + FA (0 or 400 mg/kg)	- Improved fish growth, digestive enzyme activities (protease, amylase, lipase) - Decreased FCR, SOD, CAT, GPx, and MDA levels - Increased villus height and muscular thickness in foregut	[58]
		20, 40, 80, 160 mg/kg	- Increased growth performance, hemoglobin and RBC - Decreased GLU, cortisol, SOD, CAT, GPx, and MDA levels, and phagocytic and lysozyme activities after heat stress - Upregulated the expression of INF-γ, TNF-α, and IL-1β genes - Downregulated HSP70 gene expression	[59]
	Pacific white shrimp (*Litopenaeus vannamei*)	Ferulic acid (0, 50, 100 mg/kg) or/and FA-dihydromyricetin (0, 100, 200 mg/kg)	- Increased WG, SGR, HSI, CF. digestive enzyme activities (protease, lipase, and amylase) in hepatopancreas; antioxidant enzyme activities, immune parameters - Decreased FCR, body fat content and plasma lipid parameters - Regulated the expression of genes involved in fatty acid and triglyceride metabolism in hepatopancreas - Upregulated immune-related genes in hemocytes and intestine - Modulated the composition and richness of intestinal microbiota	[60]
	Rainbow trout (*Oncorhynchus mykiss*)	10^8^ CFU *Pediocuccus pentosaceus*/g, 100 mg/kg of FA, and a combination of PP and FA	- Increased FW, WG, digestive enzyme activities (trypsin, chymotrypsin, protease, alpha-amylase, and lipase), and levels of TP, TOAC, and TC - Decrease FCR and TG - Upregulated GHRL, GHR, IGF-1, and IGF-II expression	[61]
	River prawn (*Macrobrachium nipponense*)	20, 40, 80, 160, 320 mg/kg	- Increased WGR, SGR, albumin/globulin ratio and C3; GLU, SOD, GPx - Decreased FCR, AST, iNOS, NO, MDA - Down-regulated expression of Toll and Dorsal, immune deficiency, relish, and HSP70 - Enhanced resistance against non-O1 *Vibrio cholera* GXFL1-X infection	[62]
		3% oxidized fish oil + 160 and 320 mg/kg of FA	- Increased total bile acids - Alleviated lipid droplets - Increased the abundance of *Ruminococcaceae* UCG-005 and *Lachnospiraceae*	[63]
	Wuchang bream (*Megalobrama amblycephala*)	Oxidized soybean oil + 0.06% FA + probiotics	- Promoted the accumulation of umami nucleotides (GMP, IMP, and off-flavor FAAs)	[64]
	Yellow croaker (*Larimichthys* *crocea*) larvae	20, 40, 80 mg/kg	- Higher survival rate and SGR - Increased activities of trypsin in pancreatic segments and intestinal segments, lipase in PS and alkaline phosphatase in brush border membrane; SOD and CAT - Reduced MDA and TG - Downregulated expression of lipogenesis-related genes (*SCD1*, *FAS* and *DGAT2*) - Upregulated expression of lipid catabolism-related genes (*ACO*, *CPT-1* and *HL*)	[65]
Rosmarinic acid	Common carp (*Cyprinus carpio*)	5 × 10^9^ CFU *Bacillus subtilis*/g feed, 600 mg rosmarinic acid/kg feed, combination of these additives	- Improved growth rates, digestive enzyme activities (chymotrypsin, lipase, and protease), serum immunological parameters (lysozyme, protein, ACH50, and total immunoglobulin) - Improved antioxidant-related enzyme activities in blood serum (decreasing MDA level, and improving GPX, CAT, and SOD activities) - Decreased mortality after challenging with *Aeromonas hydrophila*	[66]
	Goldfish (*Carassius auratus*)	400, 600, 800 mg/kg	- Improved growth performance - Enhanced the expression of genes, which are responsible for modulating immunity and anti-inflammatory status in a dose-dependent manner	[67]
	Rainbow trout (*Oncorhynchus mykiss*)	1.5 or 3 × 10^8^ *Lactobacillus rhamnosus*, 1 or 3 g RS/kg, and combination of both elements	- Increased FW, WG, SGR, digestive enzyme activities (amylase, protease, and lipase), Ig content, and activities of ACH50, Ig, NBT, MPO, C3, C4, SOD, CAT, and GPx - Decreased FCR and activities of GLU, ALT, ALP, LDH, and cortisol - Higher values of lysozyme, ACH50, Ig, NBT, MPO, C3, C4, SOD, CAT, and GPx after ammonia exposure - Lower levels of MDA, GLU, cortisol, ALT, ALP, and LDH after ammonia exposure	[68]

A list of abbreviations is included at the end of the paper.

**Table 2:** Caffeic acid concentration in various plant sources and by-products.

Source/By-Product	CA Concentration (mg/g)	References
**Fruits**		
Wild cherry	10.00–12.00	[74]
Coconut (*Cocos nucifera*) fruit	0.0485–2.231	[75]
Earleaf acacia (*Acacia auriculiformis*)	0.0485–2.231	[75]
Emblica officinalis fruits	0.0485–2.231	[75]
Mulberry	0.200–0.570	[69]
Quince	0.200–0.570	[69]
Sweet granadilla (*Passiflora ligularis*)	0.200–0.570	[69]
Rowanberry	0.59–0.96	[72]
Chokeberry	0.59–0.96	[72]
Sweet rowanberry	0.59–0.96	[72]
Saskatoon berry	0.59–0.96	[72]
Blueberry	0.59–0.96	[72]
Blueberries	0.20–1.00	[76]
Apple (*Valkea kuulas*)	0.28	[72]
Dark plum (*Syzygium cumini*)	0.28	[72]
Plums	0.005–0.02	[76]
Kiwis	0.005–0.02	[76]
**Coffee and related products**		
Green coffee beans (dw)	49.57	[70,71]
Defective coffee beans (dw)	38.00	[70,71]
Green coffee beverage (dw)	33.70	[77]
Coffee canephora seeds	12.33	[78]
Coffee pulp (dw)	2.00	[71]
Cascara (dw)	1.10	[70,71]
Coffee husk (dw)	0.839	[70,71]
**Teas and Other Sources**		
Green/black teas	0.30–0.36	[72]

dw, dried weight.

**Table 3:** Summary of caffeic acid and its derivatives’ effects in pathogenic microorganisms.

Compound	Pathogens	Effective Concentrations	References
Caffeic acid	*Aspergillus brasiliensis*	- Inhibited 100% *A. brasiliensis* growth at 20 mmol/dm^3^	[113]
	*Aspergillus flavus*	- Inhibited the growth of *A. flavus* at 0.2 mg/mL	[124]
	*Aspergillus parasiticus*	- Inhibited the growth of *A. parasitius* at 0.2 mg/mL	[124]
	*Bacillus cereus*	- Inhibited the growth of *B. cereus* at 0.5 mg/mL - MIC value was 100 μM	[124,125]
	*Candida albicans*	- Inhibited 100% *C. albicans* growth at 1.25 mmol/dm^3^	[113]
	Canine distemper virus	- IC_50_ at 1 and 2 h post infection was 23.3 and 32.3 μg/mL - Synergistic effect with Ribavirin against the virus - Reduced the total viral RNA synthesis by 59–86% at 24–72 h	[126]
	*Citrobacter freundii*	- IC_50_ value ranged from 200–400 μg/mL - Inhibition zone was 7.2 mm	[110,127]
	*Cladosporium* sp.	- Inhibition of 7.2% *Cladosporium* sp. growth	[128]
	*Clostridium botulinum*	- Inhibited germination of *C. botulinum* at 0.78 mM for 6 h - Reduced 80 °C spore thermal resistance at 50 mM	[129]
	*Colletotrichum* sp.	- Inhibition of 15% *Colletotrichum* sp. growth	[128]
	*Enterobacter aerogenes*	- IC_50_ value ranged from 200–400 μg/mL - Inhibition zone was 8.4 mm	[110,127]
	*Enterobacter cloacae*	- IC_50_ value ranged from 200–400 μg/mL - Inhibition zone was 10 mm	[110,127]
	*Enterococcus faecalis*	- MIC value was 100 μM	[125]
	*Escherichia coli*	- Indifference effect with streptomycin - Inhibited 100% *E. coli* growth at 5 mmol/dm^3^ - Inhibition zone ranged from 9–10.3 mm - IC_50_ value ranged from 200–400 μg/mL - MIC value was 1 mg/mL - Synergistic effect with imipenem	[113,114,120,127,130]
	*Fusarium graminearum*	- IC_50_ ranged from 6.7–10 mM	[131]
	*Fusarium oxysporum*	- Inhibition of 100% *F. oxysporum* growth	[128]
	Hepatitis C virus	- Modulated Keap1/Nrf2 interaction via increasing p62 expression, leading to stabilization of Nrf2 and HO-1 induction - Elicited IFN-α antiviral response to suppress virus replication	[126]
	Herpes Simplex Virus type 1	- Inhibited the multiplication of HSV-1 from 4–8 mM	[132]
	Influenza A virus	- Suppressed (at least temporally) the degeneration of the virus-infected cells at 4 mM	[133]
	*Klebsiella oxytoca*	- IC_50_ value ranged from 200–400 μg/mL - MIC value was 1 mg/mL	[127,130]
	*Klebsiella pneumoniae*	- Inhibited the growth of *K. pneumoniae* at 0.3–0.5 mg/mL - MIC and MBC values were 5 mg/mL	[113,124]
	*Methicillin-resistant Staphylococcus aureus*	- MIC value was 52–1024 μg/mL - Indifference effect with minocycline, vancomycin, ofloxacin, and rifampicin	[130,134]
	*Methicillin-susceptible Staphylococcus aureus*	- MIC value was 1 mg/mL	[130]
	*Mucor* sp.	- Inhibition of 10.6% *Mucor* sp. growth	[128]
	*Proteus hauseri*	- IC_50_ value ranged from 200–400 μg/mL - MIC value was 1 mg/mL	[127,130]
	*Proteus mirabilis*	- IC_50_ value ranged from 200–400 μg/mL - MIC value was 1 mg/mL	[127,130]
	*Pseudomonas aeruginosa*	- Inhibited 100% *P. aeruginosa* growth at 5 mmol/dm^3^ - MIC was 31.3 μg/mL - Synergistic effect with erythromycin, rifampicin, gentamycin and imipenem	[113,114,135]
	*Pythium* sp.	- Inhibition of 16.7% *Pythium* sp. growth	[128]
	*Rhizoctonia solani*	- Inhibition of 27.3% *Rhizoctonia solani* growth	[128]
	*Rhizopus* sp.	- Inhibition of 54.2% *Rhizopus* sp. growth	[128]
	*Salmonella enterica*	- IC_50_ value ranged from 200–400 μg/mL - MIC value was 1 mg/mL	[127,130]
	*Serratia marcescens*	- MIC value was 1 mg/mL	[130]
	*Staphylococcus aureus*	- Antagonism effect with streptomycin - Inhibited 80% biofilm at 4 μg/mL - Inhibited 100% *S. aureus* growth at 5 mmol/dm^3^ - Inhibition zone > 12 mm - MBC was 1.25 mg/mL - MIC ranged from 62.5–> 1024 μg/mL - Synergistics effect with norfloxacin	[111,112,113,114,117,119,120,125]
	*Staphylococcus epidermidis*	- MBC was 0.625 mg/mL - MIC ranged from 0.625–1 mg/mL	[119,130]
	Thrombocytopenia syndrome virus	- IC_50_ value was 0.048 mM	[136]
	*Verticillium* sp.	- Inhibition of 27.4% *Verticillium* sp. growth	[128]
Caffeic acid-amides	*Bacillus subtilis*	- MIC ranged from 1.18–15.5 μg/mL based on each amide	[137]
Caffeic acid-alkyl esters	*Aspergillus flavus*	- MIC value was 100 μg/mL	[138]
	*Bacillus cereus*	- MIC ranged from under 1.25–10 mM	[139]
	*Candida albicans*	- MIC value was 7.81–250 μg/mL - MFC ranged from 7.81–125 μg/mL - Propyl caffeate showed synergistic with fluconazole and nystatin	[138,140]
	*Escherichia coli*	- MIC ranged from 0.1–10 mM	[139,141]
	*Fusarium culmorum*	- MIC ranged from under 1.25–10 mM	[139]
	Hepatitis C virus	- EC_50_ values ranged from 1–109.6 μM	[142]
	*Proteus vulgaris*	- MIC value was 50 μg/mL	[138]
	*Saccharomyces cerevisiae*	- MIC ranged from 1–> 20 mM	[139]
	*Staphylococcus aureus*	- MIC ranged from 0.1–0.16 mM	[141]
	*Trichophyton mentagrophytes*	- MIC value was 100 μg/mL	[138]
Caffeic acid-ester derivatives	*Alternaria alternata*	- MIC ranged from 25–> 50 μg/mL	[143]
	*Candida albicans*	- MIC_50_ ranged from 16–256 μg/mL for biofilm inhibition - MIC_50_ ranged from 2–128 μg/mL for planktonic cells	[144]
	*Colletotrichum truncatum*	- MIC ranged from 12.5–> 50 μg/mL	[143]
	*Escherichia coli*	- MIC ranged from 0.20–1.31 μM	[145]
	*Fusarium equiseti*	- MIC ranged from 25–> 50 μg/mL	[143]
	*Fusarium graminearum*	- MIC ranged from 12.5–> 50 μg/mL	[143]
	*Paenibacillus larvae*	- MIC ranged from 125–> 500 μg/mL	[146]
	*Phomopsis longicolla*	- MIC ranged from 3.25–> 50 μg/mL	[143]
	*Septoria bataticola*	- MIC value > 50 μg/mL	[143]
	*Staphylococcus aureus*	- MIC ranged from 0.21–1.41 μM	[145]
Caffeic acid-N-nonyl ester	*Agrobacterium tumefaciens*	- MIC value was > 100 mM	[147]
	*Bacillus subtilis*	- Inhibition zone was > 12 mm - MBC and MIC value was 10 mM	
	*Escherichia coli*	- Inhibition zone ranged from 10–12 mm - MBC and MIC value was 10 mM	
	*Klebsiella rhinoscleromatis*	- Inhibition zone ranged from 10–12 mm - MBC and MIC value was 100 mM	
	*Pseudomonas aeruginosa*	- MIC value was > 100 mM	
	*Salmonella* sp.	- Inhibition zone ranged from 10–12 mm - MBC and MIC value was 10 mM	
	*Staphylococcus aureus*	- Inhibition zone ranged from 10–12 mm - MBC and MIC value was 10 mM	
Caffeic acid nanoparticles	*Ralstonia solanacearum*	- EC_50_ value was 0.285 mg/mL	[148]
Caffeic acid-phenethyl ester	*Actinomyces viscosus*	- MBC ranged from 0.32–4 mg/mL - MIC ranged from 0.16–2 mg/mL	[149]
	*Aspergillus niger*	- MIC value > 1.8 mM	[150]
	*Bacillus subtilis*	- MIC ranged from 0.44–0.71 mM	[150]
	*Bacillus megaterium*	- MIC value was 50 mM	[151]
	*Candida albicans*	- MIC ranged from 0.88–2.8 mM	[150]
	Influenza virus type A and B	- Inhibited 92–95% virus growth at 8 mM	[150]
	*Klebsiella* spp.	- MIC value was 50 mM	[151]
	*Lactobacillus acidophilus*	- MBC ranged from 0.32–8 mg/mL - MIC ranged from 0.16–4 mg/mL	[149]
	*Pseudomonas aeruginosa*	- MIC ranged from 0.88–1.4 mM	[150]
	*Staphylococcus aureus*	- MIC ranged from 0.44–1.4 mM	[150]
	*Streptococcus olysgalactiae*	- MIC value was 48 mM	[151]
	*Streptococcus mitis*	- MIC value was 55 mM	[151]
	*Streptococcus mutans*	- MBC ranged from 0.32–4 mg/mL - MIC ranged from 0.16–2 mg/mL	[149]
	*Streptococcus sobrinus*	- MBC ranged from 0.32–16 mg/mL - MIC ranged from 0.16–8 mg/mL	[149]

## Data Availability

No new data were created or analyzed in this study.

## Abbreviations

The following abbreviations are used in this manuscript:

ACH_50_	Alternative Complement Hemolytic 50 Activity
ALB	Albumin
ALP	Alkaline Phosphatase
ALT	Alanine Aminotransferase
AST	Aspartate Aminotransferase
AVM	Abamectin
BUN	Blood Urea Nitrogen
CA	Caffeic Acid
CAPA	Caffeic Acid Phenethyl Amide
CAPE	Caffeic Acid Phenethyl Ester
CAT	Catalase
CF	Condition Factor
CGA	Chlorogenic Acid
CS-g-CA	Chitosan-grafted Caffeic Acid
DAO DAMPs	Diamine Oxidase Damage-associated molecular patterns
ET-1	Endothelin-1
FA	Ferulic Acid
FCR	Feed Conversion Ratio
FER	Feed Efficiency Ratio
FBW	Final Body Weight
FI	Feed Intake
FW	Final Weight
GLO	Globulin
GLU	Glucose
GPx	Glutathione Peroxidase
GSH	Glutathione
GSH-Px	Glutathione Peroxidase
GST	Glutathione S-Transferase
HDL	High-Density Lipoprotein
HFD	High-Fat Diet
HO-1	Heme Oxygenase-1
Ig	Immunoglobulin
IgM	Immunoglobulin M
IL	Interleukin
Keap1	Kelch-like ECH-associated protein 1
LD	Lactate Dehydrogenase
LDH	Lactate Dehydrogenase
LDLC	Low-Density Lipoprotein Cholesterol
LPS	Lipopolysaccharide
LYM	Lymphocyte
LYZ	Lysozyme
MDA	Malondialdehyde
MPO	Myeloperoxidase
MTs	Metallothioneins
NF-κB	Nuclear Factor kappa-light-chain-enhancer of activated B cells
NFI-3 NLRs	Nuclear Factor Interleukin-3 NOD-like receptors
NLRP3	NOD-, LRR- and pyrin domain-containing protein 3
Nrf2	Nuclear Factor Erythroid 2-related Factor 2
PAMPs	Pathogen-Associated Molecular Patterns
PER	Protein Efficiency Ratio
PP	Pediococcus pentosaceus
PRRs	Pattern Recognition Receptors
PPARγ	Peroxisome Proliferator-Activated Receptor Gamma
ROS	Reactive Oxygen Species
RA	Rosmarinic Acid
RLRs	RIG-I-Like Receptors
SGR	Specific Growth Rate
SOD	Superoxide Dismutase
SNP	Sodium Nitroprusside
TAS	Total Antioxidant Status
TC	Total Cholesterol
TGs	Triglycerides
TGF-β	Transforming Growth Factor Beta
TLR	Toll-Like Receptor
TP	Total Protein
WG	Weight Gain
WGR	Weight Gain Rate
WSSV	White Spot Syndrome Virus
ZO-1	Zonula Occludens-1
